# Supplementary material for: Comparative Analysis of Aerotolerance, Antibiotic Resistance, and Virulence Gene Prevalence in Campylobacter jejuni Isolates from Retail Raw Chicken and Duck Meat in South Korea
Source: Microorganisms. 2019 Oct 10;7(10):433. doi: 10.3390/microorganisms7100433 (PMC6843641; doi:10.3390/microorganisms7100433)
Supplement: Supplementary file 1 [file microorganisms-07-00433-s001.pdf]

Table S1. Prevalence of virulence genes in oxygen-sensitive (OS), aerotolerant (AT), and hyper-aerotolerant (HAT) *C. jejuni* strains from retail raw chicken and duck meat

**Virulence gene**	**No. (%) of positive strains**					
	**Chicken**			**Duck**		
	**OS (n=12)**	**AT (n=45)**	**HAT (n=33)**	**OS (n=6)**	**AT (n=28)**	**HAT (n=11)**
***cad*F**	12 (100.0)	45 (100.0)	32 (97.0)	4 (66.7)	28 (100.0)[a]	10 (90.9)
***cdt*B**	12 (100.0)	44 (97.8)	32 (97.0)	5 (83.3)	20 (71.4)	10 (90.9)
iam	12 (100.0)	44 (97.8)	32 (97.0)	3 (50.0)	28 (100.0)[b]	9 (81.8)
***pld*A**	11 (91.7)	43 (95.6)	31 (93.9)	4 (66.7)	28 (100.0)[a]	9 (81.8)
***vir*B11**	2 (16.7)	3 (6.7)	2 (6.1)	1 (16.7)	2 (7.1)	0 (0.0)
***doc*A**	12 (100.0)	45 (100.0)	31 (93.9)	5 (83.3)	28 (100.0)[a]	11 (100.0)
***peb*1**	12 (100.0)	41 (91.1)	31 (93.9)	4 (66.7)	27 (96.4)[a]	10 (90.9)
***fla*A**	12 (100.0)	45 (100.0)	33 (100.0)	5 (83.3)	27 (96.4)	10 (90.9)
***cia*B**	12 (100.0)	44 (97.8)	30 (90.9)	5 (83.3)	27 (96.4)	8 (72.7)

[a] Indicates AT (duck) significantly different from OS (duck) ($p<0.05$).
[b] Indicates AT (duck) significantly different from OS (duck) ($p<0.0001$).

Table S2. Distribution of *C. jejuni* strains belonging to different Viro & AMR (virulence and antibiotic resistance) types depending on the aerotolerance, source (chicken and duck meat), and season (summer and winter)

Type	No. (%) of strains						
	Aerotolerance			Sample		Season	
	OS (n=18)	AT (n=73)	HAT (n=44)	Chicken (n=90)	Duck (n=45)	Summer (n=88)	Winter (n=47)
Type 01	1 (5.6)	1 (1.4)	1 (2.3)	2 (2.2)	1 (2.2)	2 (2.3)	1 (2.1)
Type 02	1 (5.6)	3 (4.1)	1 (2.3)	5 (5.6)	0 (0.0)	2 (2.3)	3 (6.4)
Type 03	0 (0.0)	1 (1.4)	0 (0.0)	0 (0.0)	1 (2.2)	0 (0.0)	1 (2.1)
Type 04	5 (27.8)	26 (35.6)	18 (40.9)	38 (42.2)	11 (24.4)	36 (40.9)	13 (27.7)
Type 05	0 (0.0)	2 (2.7)	2 (4.5)	3 (3.3)	1 (2.2)	2 (2.3)	2 (4.3)
Type 06	0 (0.0)	0 (0.0)	1 (2.3)	1 (1.1)	0 (0.0)	1 (1.1)	0 (0.0)
Type 07	0 (0.0)	0 (0.0)	1 (2.3)	0 (0.0)	1 (2.2)	1 (1.1)	0 (0.0)
Type 08	0 (0.0)	1 (1.4)	0 (0.0)	1 (1.1)	0 (0.0)	1 (1.1)	0 (0.0)
Type 09	0 (0.0)	6 (8.2)	0 (0.0)	0 (0.0)	6 (13.3)	0 (0.0)	6 (12.8)
Type 10	0 (0.0)	3 (4.1)	0 (0.0)	1 (1.1)	2 (4.4)	0 (0.0)	3 (6.4)
Type 11	0 (0.0)	0 (0.0)	1 (2.3)	0 (0.0)	1 (2.2)	1 (1.1)	0 (0.0)
Type 12	0 (0.0)	1 (1.4)	0 (0.0)	1 (1.1)	0 (0.0)	1 (1.1)	0 (0.0)
Type 13	5 (27.8)	14 (19.2)	8 (18.2)	14 (15.6)	13 (28.9)	21 (23.9)	6 (12.8)
Type 14	1 (5.6)	1 (1.4)	0 (0.0)	2 (2.2)	0 (0.0)	0 (0.0)	2 (4.3)
Type 15	1 (5.6)	1 (1.4)	0 (0.0)	1 (1.1)	1 (2.2)	2 (2.3)	0 (0.0)
Type 16	1 (5.6)	0 (0.0)	0 (0.0)	0 (0.0)	1 (2.2)	0 (0.0)	1 (2.1)
Type 17	0 (0.0)	0 (0.0)	2 (4.5)	1 (1.1)	1 (2.2)	0 (0.0)	2 (4.3)
Type 18	0 (0.0)	1 (1.4)	0 (0.0)	1 (1.1)	0 (0.0)	0 (0.0)	1 (2.1)
Type 19	0 (0.0)	0 (0.0)	1 (2.3)	1 (1.1)	0 (0.0)	0 (0.0)	1 (2.1)
Type 20	0 (0.0)	2 (2.7)	0 (0.0)	2 (2.2)	0 (0.0)	2 (2.3)	0 (0.0)
Type 21	1 (5.6)	5 (6.8)	4 (9.1)	9 (10.0)	1 (2.2)	8 (9.1)	2 (4.3)
Type 22	0 (0.0)	2 (2.7)	0 (0.0)	2 (2.2)	0 (0.0)	2 (2.3)	0 (0.0)
Type 23	0 (0.0)	2 (2.7)	0 (0.0)	2 (2.2)	0 (0.0)	2 (2.3)	0 (0.0)
Type 24	0 (0.0)	1 (1.4)	0 (0.0)	0 (0.0)	1 (2.2)	1 (1.1)	0 (0.0)
Type 25	0 (0.0)	0 (0.0)	1 (2.3)	1 (1.1)	0 (0.0)	0 (0.0)	1 (2.1)
Type 26	0 (0.0)	0 (0.0)	1 (2.3)	1 (1.1)	0 (0.0)	0 (0.0)	1 (2.1)
Type 27	0 (0.0)	0 (0.0)	1 (2.3)	1 (1.1)	0 (0.0)	1 (1.1)	0 (0.0)
Type 28	1 (5.6)	0 (0.0)	0 (0.0)	0 (0.0)	1 (2.2)	0 (0.0)	1 (2.1)
Type 29	0 (0.0)	0 (0.0)	1 (2.3)	0 (0.0)	1 (2.2)	1 (1.1)	0 (0.0)
Type 30	1 (5.6)	0 (0.0)	0 (0.0)	0 (0.0)	1 (2.2)	1 (1.1)	0 (0.0)

Table S3. Primers used in this study

Gene	Primer	Sequence (5′-3′)	Size (bp)	Reference
16S rRNA	C412F	GGATGACACTTTTCGGAGC	816	[1]
	C1228R	CATTGTAGCACGTGTGTC		
cj0414	C-1	CAAATAAAGTTAGAGGTAGAATGT	161	[2]
	C-3	CCATAAGCACTAGCTAGCTGAT		
hipO	hipO_F	GCAAAATCCACAGCTTCATCGT	350	This study
	hipO_R	GGAAGGGGTGGTCATGGAAG		
ask	CC18F	GGTATGATTTCTACAAAGCGAG	502	[3]
	CC519R	ATAAAAGACTATCGTCGCGTG		
cadF	cadF_F	TTGAAGGTAATTTAGATATG	400	[4]
	cadF_R	CTAATACCTAAAGTTGAAAC		
cdtB	cdtB_F	GTTAAAATCCCCTGCTATCAACCA	495	[5]
	cdtB_R	GTTGGCACTTGGAATTTGCAAGGC		
ciaB	ciaB_F	TTTCCAAATTTAGATGATGC	1165	[6]
	ciaB_R	GTTCTTTAAATTTTTCATAATGC		
docA	docA_F	ATAAGGTGCGGTTTTGGC	725	[6]
	docA_R	GTCTTTGCAGTAGATATG		
iam	iamA_F	GCACAAAATATATCATTACAA	518	[4]
	iamA_R	TTCACGACTACTATGAGG		
peb1	peb1_F	TAATACGACTCACTATAGGGGAAAATCTTT	775	[7]
	peb1_R	TTTTCGCTAAAGCATCAATTTCATT		
pldA	pldA_F	AAGCTTATGCGTTTTT	913	[8]
	pldA_R	TATAAGGCTTTCTCCA		
virB11	virB11_F	GAACAGGAAGTGGAAAAACTAGC	708	[9]
	virB11_R	TTCCGCATTGGGCTATATG		
flaA	flaA_F	ATGGGATTTCGTATTAACAC	1713	[6]
	flaA_R	CTGTAGTAATCTTAAAACATTTTG		

References

1. Linton, D.; Owen, R.J.; Stanley, J. Rapid identification by PCR of the genus *Campylobacter* and of five *Campylobacter* species enteropathogenic for man and animals. *Res. Microbiol.* **1996**, *147*, 707-718.
2. Wang, R.F.; Slavic, M.F.; Cao, W.W. A rapid PCR method for direct detection of low numbers of *Campylobacter jejuni*. *J. Rapid. Methods Autom. Microbiol.* **1992**, *1*, 101–108.

3. Linton, D.; Lawson, A.J.; Owen, R.J.; Stanley, J. PCR detection, identification to species level, and fingerprinting of *Campylobacter jejuni* and *Campylobacter coli* direct from diarrheic samples. *J. Clin. Microbiol.* **1997**, *35*, 2568-2572.
4. Konkel, M.E.; Gray, S.A.; Kim, B.J.; Garvis, S.G.; Yoon, J. Identification of the enteropathogens *Campylobacter jejuni* and *Campylobacter coli* based on the cadf virulence gene and its product. *J. Clin. Microbiol.* **1999**, *37*, 510-517.
5. Bang, D.D.; Scheutz, F.; Ahrens, P.; Pedersen, K.; Blom, J.; Madsen, M. Prevalence of cytolethal distending toxin (*cdt*) genes and cdt production in *Campylobacter* spp. isolated from danish broilers. *J. Med. Microbiol.* **2001**, *50*, 1087-1094.
6. Muller, J.; Schulze, F.; Muller, W.; Hanel, I. PCR detection of virulence-associated genes in *Campylobacter jejuni* strains with differential ability to invade caco-2 cells and to colonize the chick gut. *Vet.Microbiol.* **2006**, *113*, 123-129.
7. Biswas, D.; Hannon, S.J.; Townsend, H.G.; Potter, A.; Allan, B.J. Genes coding for virulence determinants of *Campylobacter jejuni* in human clinical and cattle isolates from Alberta, Canada, and their potential role in colonization of poultry. *Int. Microbiol.* **2011**, *14*, 25-32.
8. Datta, S.; Niwa, H.; Itoh, K. Prevalence of 11 pathogenic genes of *Campylobacter jejuni* by PCR in strains isolated from humans, poultry meat and broiler and bovine faeces. *J. Med. Microbiol.* **2003**, *52*, 345-348.
9. Bacon, D.J.; Alm, R.A.; Hu, L.; Hickey, T.E.; Ewing, C.P.; Batchelor, R.A.; Trust, T.J.; Guerry, P. DNA sequence and mutational analyses of the pVir plasmid of *Campylobacter jejuni* 81-176. *Infect. Immun.* **2002**, *70*, 6242-6250.

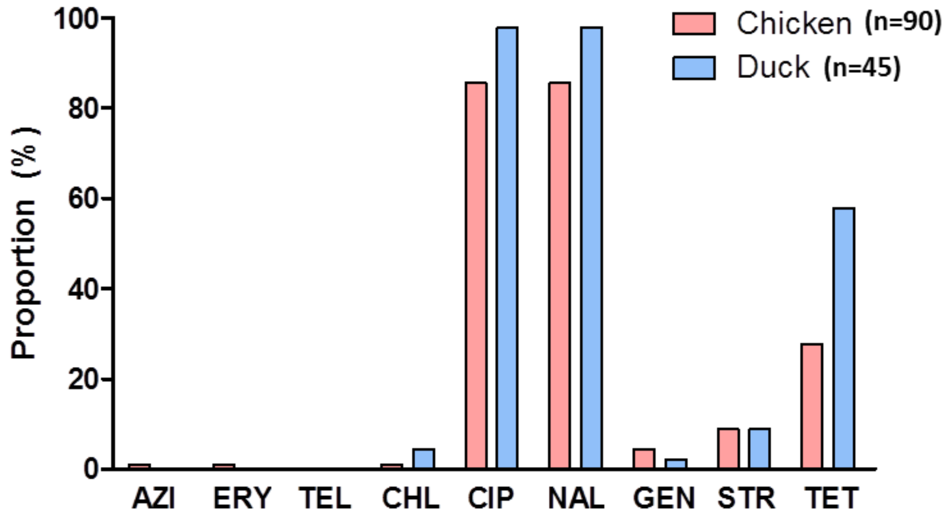

Fig. S1. Antibiotic resistance in *C. jejuni* strains from retail raw chicken and duck meat.

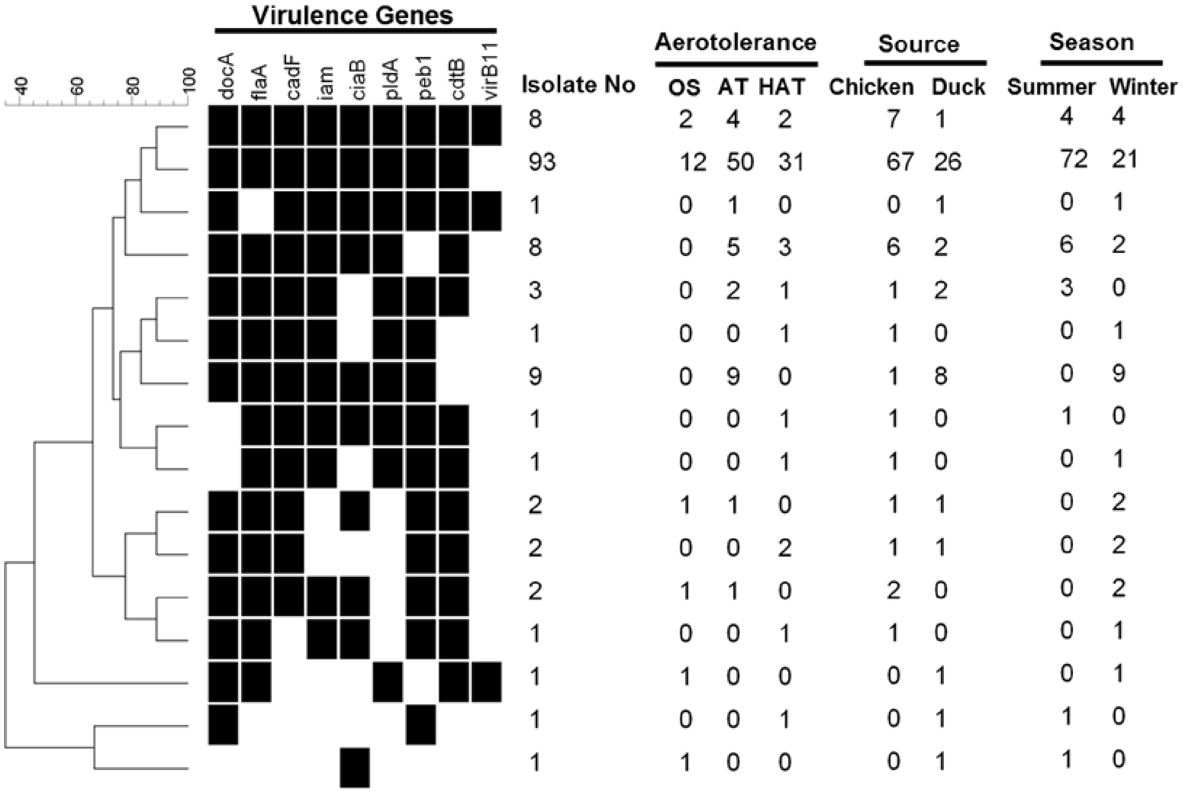

Fig. S2. Prevalence of nine virulence genes in 135 strains of *C. jejuni* from retail raw chicken and duck meats in Korea. The number of strains is indicated on right.
